# Revolutionary self-powered transducing mechanism for long-lasting and stable glucose monitoring: achieving selective and sensitive bacterial endospore germination in microengineered paper-based platforms

**DOI:** 10.1038/s41378-024-00836-9

**Published:** 2024-12-12

**Authors:** Yang Gao, Anwar Elhadad, Seokheun Choi

**Affiliations:** 1 Department of Electrical & Computer Engineering, Bioelectronics & Microsystems Laboratory, State University of New York at Binghamton, Binghamton, NY 13902 USA; 2 Center for Research in Advanced Sensing Technologies & Environmental Sustainability, State University of New York at Binghamton, Binghamton, NY 13902 USA

**Keywords:** Electrical and electronic engineering, Materials science

## Abstract

We introduce a groundbreaking proof-of-concept for a novel glucose monitoring transducing mechanism, marking the first demonstration of a spore-forming microbial whole-cell sensing platform. The approach uses selective and sensitive germination of *Bacillus subtilis* spores in response to glucose in potassium-rich bodily fluids such as sweat. As the rate of germination and the number of metabolically active germinating cells are directly proportional to glucose concentration, the electrogenic activity of these cells—manifested as electricity—serves as a self-powered transducing signal for glucose detection. Within a microengineered, paper-based microbial fuel cell (MFC), these electrical power outputs are measurable and can be visually displayed through a compact interface, providing real-time alerts. The dormant spores extend shelf-life, and the self-replicating bacteria ensure robustness. The MFC demonstrated a remarkable sensitivity of 2.246 µW·(log mM)^−1^·cm^−2^ to glucose concentrations ranging from 0.2 to 10 mM, with a notably lower limit of detection at ~0.07 mM. The sensor exhibited exceptional selectivity, accurately detecting glucose even in the presence of various interferents. Comparative analyses revealed that, unlike conventional enzymatic biosensors whose performance degrades significantly through time even when inactive, the spore-based MFC is stable for extended periods and promptly regains functionality when needed. This preliminary investigation indicates that the spore-forming microbial whole-cell sensing strategy holds considerable promise for efficient diabetes management and can be extended toward noninvasive wearable monitoring, overcoming critical challenges of current technologies and paving the way for advanced biosensing applications.

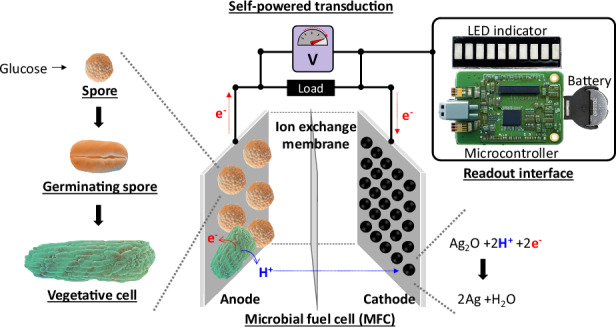

## Introduction

Diabetes is a chronic metabolic disorder characterized by elevated blood glucose levels, leading to severe complications such as cardiovascular diseases, blood vessel damage, diabetic retinopathy, kidney failure, and neuropathy^1,2^. These complications can result in significant health issues and even death if not properly managed. Type 1 diabetes, an autoimmune condition requiring stringent glucose monitoring and regular insulin injections, accounts for a small percentage of diabetes cases^3^. In contrast, more than 90% of individuals with diabetes have Type 2 diabetes, which can be effectively managed or delayed through regular blood glucose monitoring to prevent complications and ensure optimal health outcomes^4^. With the prevalence of Type 2 diabetes projected to double from 529 million people in 2021 to at least 1.3 billion by 2050 worldwide, the importance of effective glucose monitoring is becoming increasingly critical^4,5^. Although current medical treatments cannot cure diabetes, glucose monitoring helps identify patterns, achieve target ranges, and ultimately improve health outcomes^6,7^. It enables patients to understand how factors such as sleep, diet, exercise, and medications affect their blood glucose levels. Given these needs, the trend in glucose monitoring is shifting from traditional single-use devices to continuous and non-invasive monitoring systems^8,9^. Continuous glucose monitoring (CGM) systems offer real-time data and a comprehensive understanding of glucose levels through time. Unlike traditional single-use devices that require multiple daily finger pricks, CGMs provide continuous feedback on glucose trends and patterns, empowering users to make more informed health management decisions. Numerous CGM systems, which non-invasively measure glucose levels in interstitial fluid, have received FDA approval and are leading the market^10^. Meanwhile, alternative biofluids such as tears, saliva, and sweat are being intensively explored for the next generation of CGM technologies^9,10^. This trend underscores the growing importance and market expansion of continuous and long-term glucose monitoring technologies in effectively managing diabetes, offering greater convenience, improved glucose control, and potentially better long-term health outcomes.

However, even the latest commercial CGM systems are limited to a lifespan of several days and require specific storage conditions to maintain their shelf life^11,12^. This limitation primarily stems from the reliance on enzymatic electrochemical transduction mechanisms for glucose monitoring, as enzymes tend to denature and deactivate over time^11,13^. Despite several decades of development, glucose monitoring technology has predominantly relied on three-electrode enzyme-based electrochemical techniques^14^, where the catalytic activity of glucose-specific enzymes facilitates the detection and quantification of glucose levels. These techniques have been widely adopted for point-of-care and at-home settings because of their low cost, high selectivity, portability, and user-friendly features^15^. The promise of simple, stable self-powered monitoring systems remains elusive even though electricity can be generated by the redox conversion of glucose upon a specific bonding between the glucose and the enzymes, which become unstable through time^16–18^. While emerging techniques in enzyme immobilization, protein engineering, and chemical modification have demonstrated the potential to prolong enzymatic activity and stability^14,15^, these advances have not achieved the necessary breakthroughs to fully resolve issues related to enzyme denaturation and instability. Recently, non-enzymatic electrochemical glucose sensors, which use electrocatalytically active materials to directly catalyze glucose, have offered a promising solution to these stability issues^12,19^. Nevertheless, their low selectivity and performance have prevented them from practical demonstrations^20,21^.

**Fig. 1:**
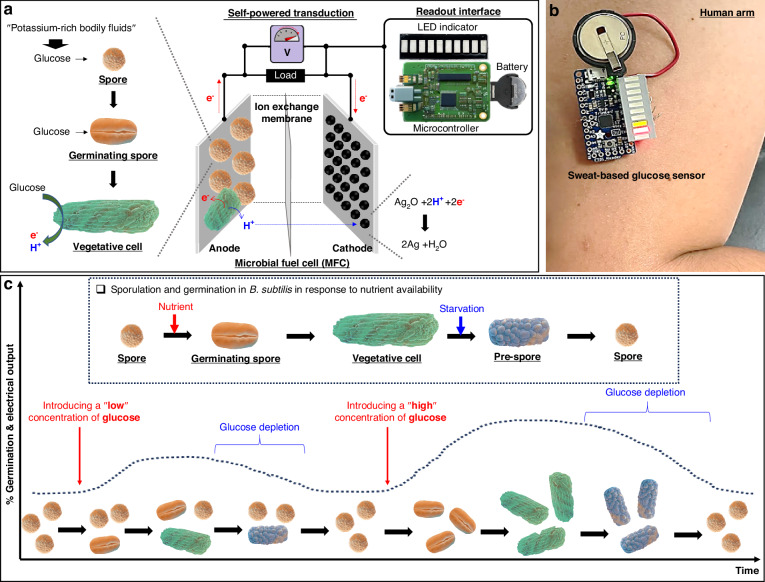
**A depiction of a paper-based, spore-forming microbial whole-cell sensing system developed for glucose monitoring**. **a** Selective and sensitive germination of *Bacillus subtilis* spores in response to glucose present in potassium-rich bodily fluids within a microbial fuel cell (MFC). The rate of germination and the number of metabolically active cells are directly proportional to the glucose concentration. As these cells become active, they generate electricity, which powers a transducing signal for glucose detection. The electrical output is measurable and can be visually displayed through a compact readout interface, providing real-time alerts. **b** Photographic evidence of the wearable design of our paper-based glucose sensing system, emphasizing its practical application. **c** A schematic illustrating sporulation and germination in *B. subtilis*, demonstrating the system’s capability for long-term glucose monitoring

This study introduces a groundbreaking strategy using a spore-forming microbial whole-cell sensing system, offering a novel solution for glucose detection. Traditional whole-cell-based sensing techniques enhance enzymatic sensor stability by using living cells to self-sustainably and continuously produce necessary enzymes^22^. However, their low specificity for biomarkers such as glucose and the need to maintain cell viability have limited their application to general toxicity sensors^23^. Genetic engineering and advances in synthetic biology have provided some selectivity^24^, but these methods are stringent, expensive, and time-consuming. Moreover, recent research has not adequately addressed the challenge of maintaining cell viability as bioreceptors. Our innovative system addresses those issues by triggering spore germination in response to glucose presence and concentration, leading to metabolically active cells that generate electricity within an engineered paper-based microbial fuel cell (MFC) (Fig. 1a, b). The dormant spores significantly extend shelf-life and ensure bacterial viability. Upon germination, the microbial ability to self-repair and self-replicate enhances the system’s robustness and longevity (Fig. 1c). The transduction mechanism specifically designed for glucose detection is self-powered and self-sustaining (Fig. 1a). Spore germination is triggered by micromolar concentrations of glucose without relying on power-intensive electrochemical signal conversion systems, and the resulting shape changes can be monitored electrically (Fig. 1c). This process uses the exoelectrogenic properties of the spore-forming bacterium *Bacillus subtilis*, which, upon germination, release metabolically generated electrons across their cell membrane to terminal electrodes. While glucose alone does not directly trigger full germination of the endospores, it acts as a co-factor in potassium-rich environments—such as those found in bodily fluids like sweat—making our proposed system particularly suitable for wearable applications. During germination, electron transfer to external acceptors, such as electrodes, becomes crucial for the bacteria’s growth, respiration, communication, and interaction with their surroundings, thereby facilitating its role in sensing and signal transduction. The electrical output serves as an accurate transducing signal for glucose monitoring, as the propensity for germination and the number of metabolically active vegetative cells directly correlate with glucose concentration (Fig. 1c). This correlation can be easily measured using a two-electrode MFC platform. The output signal is converted into a readable format by a coin battery-powered indicator circuit, which activates a light-emitting diode (LED) when the glucose concentration exceeds a predefined threshold (Fig. 1b). The novel spore-forming microbial whole-cell sensing system offers a powerful, cost-effective, stable, and sustainable approach for glucose monitoring, addressing critical limitations of current technologies and paving the way for more advanced and reliable biosensing applications.

## Results and discussion

### A novel transduction mechanism for glucose monitoring

In nature, specific bacterial species like *B. subtilis* exhibit remarkable adaptability by forming dormant and resilient endospores, allowing them to withstand harsh environmental conditions for centuries or longer^25–30^. These spores remain dormant without degradation, resuming metabolic activity and germinating into vegetative cells upon exposure to certain nutrient germinants. Traditionally, amino acids such as L-valine, L-alanine, and a combination of L-asparagine, glucose, fructose, and potassium chloride (AGFK) are the most commonly used germinants in laboratory settings to investigate bacterial germination mechanisms^25–30^. This process involves specialized germination-dedicated membrane proteins known as germinant receptors (GRs). In *B. subtilis*, the primary GRs include GerA, GerB, and GerK. GerA is responsive to L-valine and L-alanine, while GerB and GerK are simultaneously activated by AGFK^31^. Although glucose alone does not induce the germination of *B. subtilis* spores, it can facilitate germination through GerA by interacting with GerK when combined with trace amounts of potassium (K^+^), a prevalent electrolyte in bodily fluids such as sweat, tears, and gastrointestinal fluids^32,33^. Recent findings from our research group have demonstrated that *B. subtilis* spores can germinate in response to the nutrients present in sweat, without the need for those laboratory-specific germinants^34^. This discovery underscores the potential of leveraging naturally occurring biofluids to initiate bacterial germination and subsequently use endospores for nutrient detection. Additionally, the electrogenic capability of *B. subtilis* is well documented, with applications for power generation having been thoroughly demonstrated^35,36^. Notably, sporulation and germination in *B. subtilis* have been innovatively employed for long-term storage and on-demand power generation^34,37–40^. Building on these established principles, our hypothesis posits that *B. subtilis* spores possess a sensing mechanism that can selectively detect glucose in potassium-rich bodily fluids such as sweat (Fig. 1a). We propose that this detection occurs through germination, with the propensity for germination dependent on glucose concentration being transduced into electrical outputs as the metabolically active, germinated cells exhibit electrogenic activity (Fig. 1b, c).

**Fig. 2:**
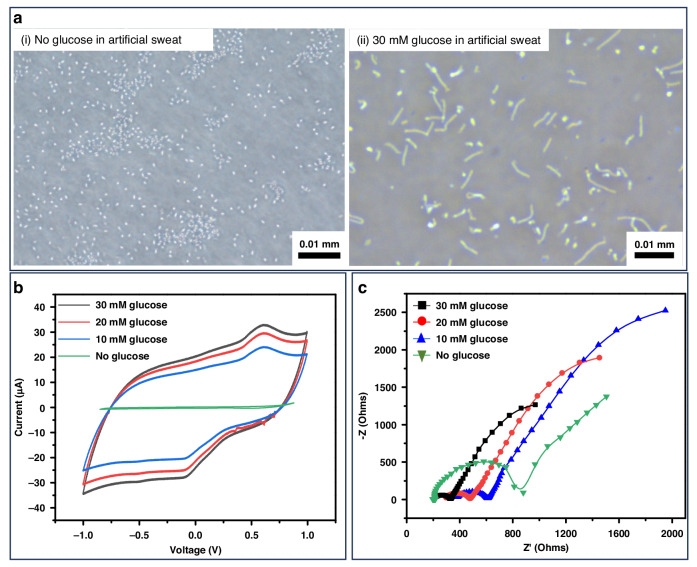
**Spore germination in response to glucose in potassium-rich artificial sweat**. **a** Comparison of spores in the absence (i) and presence (ii) of glucose, highlighting the glucose-induced germination process. **b** Cyclic voltammetry (CV) curves and (**c**) electrochemical impedance spectroscopy (EIS) data under varying glucose concentrations, providing insights into the spore’s electrochemical response and its correlation with glucose levels

To test our hypothesis, we prepared *B. subtilis* endospores in a vial and treated them with a potassium-rich artificial sweat solution, either with or without glucose (Fig. 2). Following treatment, the bacterial cells were harvested for further microbial analysis. In the absence of glucose, no germination was observed (Fig. 2a i). In contrast, the sample treated with 30 mM glucose showed clear evidence of spore germination. (Fig. 2a ii). Over four hours, we observed the spores transitioning from round-shaped spores to rod-shaped vegetative cells, indicating their ripening and outgrowth. Although full shape transformation requires time, the germination began immediately^38^, even at a low glucose concentration of 0.2 mM (Fig. S1). Fluorescent microscopy allowed us to characterize this early phase of germination. Using 5(6)-carboxyfluorescein diacetate (cFDA), germinating spores were identified by their green fluorescence, while propidium iodide (PI) stained dead cells and spores red^39^. Even before the shape change was fully completed (Fig. S1a), the round-shaped cells exhibited predominantly green fluorescence, indicating active germination, with fewer red-stained areas (Fig. S1b). Dormant endospores are not inherently electroactive; however, upon germination in response to glucose, the resulting vegetative cells become metabolically active and exhibit electrogenic properties. This metabolic activity drives electron transfer, which can be observed as redox peaks. To confirm this, we conducted cyclic voltammetry (CV) measurements using a three-electrode electrochemical cell at a scan rate of 100 mV s⁻¹. These measurements revealed notable electrochemical activity in the glucose-containing artificial sweat sample (Fig. 2b). This activity confirmed that the spores had germinated into vegetative cells and resumed metabolic processes. In the presence of glucose, *B. subtilis* displayed discernible redox peaks, with electrochemical activity increasing in correlation with glucose concentration. Conversely, in the absence of glucose, no discernible electrochemical behavior was detected, and no redox peaks were observed. Further analysis using electrochemical impedance spectroscopy (EIS) demonstrated that the introduction of glucose significantly enhanced electrochemical activities, particularly improving the electron transfer efficiency of *B. subtilis* through their germination and subsequent metabolic activity (Fig. 2c). By fitting the EIS data to the Randles equivalent circuit model, we extracted the charge transfer resistance (*R*_*ct*_) values at varying glucose concentrations. The EIS data were analyzed using ZView software, and *R*_*ct*_ values were calculated across a glucose concentration range of 0.2 mM to 10 mM. The analysis revealed a clear inverse relationship between glucose concentration and *R*_*ct*_, as shown in Table S1. Without glucose, the spores exhibited the highest charge transfer resistance, indicating a lack of metabolic redox reactions. As glucose concentration increased, the charge transfer resistance decreased, reflecting an increase in germinated cells and a corresponding enhancement in electron transfer efficiency at the electrode interface because of their metabolic activities.

**Fig. 3:**
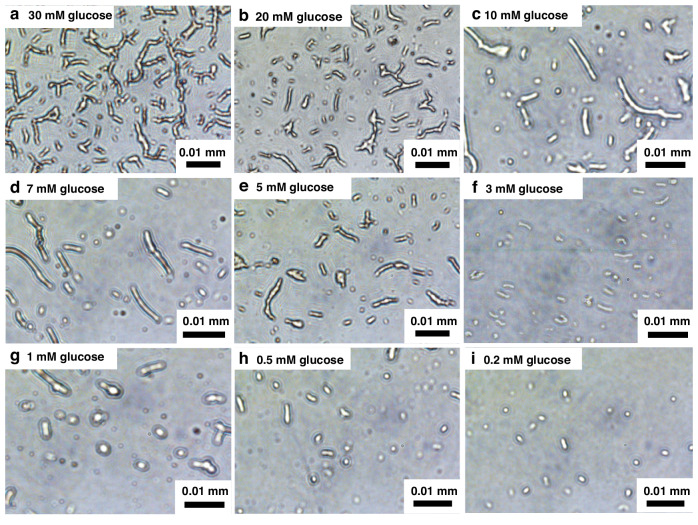
**Visual observation of spore germination across a range of glucose concentrations from 0.2** **to 30** **mM**. The specific glucose concentrations tested are as follows: (**a**) 30 mM, (**b**) 20 mM, (**c**) 10 mM, (**d**) 7 mM, (**e**) 5 mM, (**f**) 3 mM, (**g**) 1 mM, (**h**) 0.5 mM, and (**i**) 0.2 mM

After a four-hour incubation with varying glucose concentrations, we optically visualized the shape changes of the spores (Fig. 3). The likelihood of spore germination, as well as the speed of ripening and outgrowth, increased with higher glucose concentrations. At elevated glucose levels, a greater number of long rod-shaped cells were observed, indicating successful germination and transition to vegetative cells. In contrast, at lower glucose concentrations (0.2–1.0 mM), many spores retained their round shape, suggesting incomplete transformation. However, as shown in Fig. S1, even at low glucose concentrations, germination had already initiated, with spores exhibiting early signs of metabolic activity. This early-stage activity highlights the sensitivity of *B. subtilis* spores to glucose availability and suggests that even minimal glucose presence can start germination. Those observations underscore the critical role of glucose concentration in driving the morphological transformation of spores and in regulating their metabolic activation. This relationship between glucose concentration and germination efficiency offers valuable insights into the optimal conditions for monitoring glucose levels in bodily fluids. These findings have significant implications for biological research and practical applications in biosensing technologies.

### MFC-enabled self-powered glucose monitoring

In an MFC platform, germinating cells generate an electrochemical potential difference between the anode and cathode that directly correlates with glucose concentration (Fig. 1a)^37–40^. The MFC operates with three essential components: an anode containing spore-forming bacteria, an ion-exchange membrane, and a catalyst-coated cathode^41^. When glucose is present in potassium-rich bodily fluids, it fuels the germination and metabolism of the spores, triggering a redox reaction that generates electrons and protons. Electrons flow through an external circuit with a resistor, while protons pass through the internal ion-exchange membrane. At the cathode, the catalytic reaction combines the electrons and protons, completing the circuit. The number of harvested electrons and protons, derived from their electrochemical metabolic energy conversion of the germinated vegetative cells, is proportional to the glucose concentration. As the resulting electrical current passes through the resistor, voltage drops and power is produced, which can be used as a self-powered signal for glucose monitoring.

**Fig. 4:**
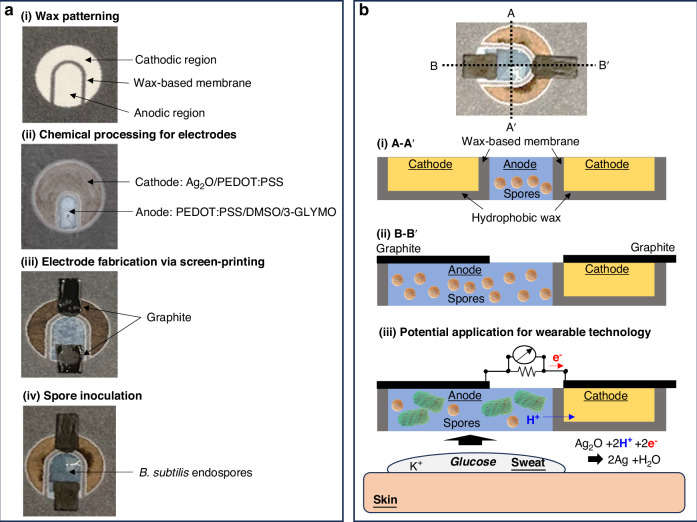
**Fabrication, configuration, and operation of the paper-based MFC glucose sensor**. **a** Fabrication process of the horizontally structured MFC on paper. **b** (i) A-A’ and (ii) B-B’ cross sectional illustration of the MFC, highlighting its configuration and (iii) potential use as a wearable device for non-invasive glucose monitoring

In this study, we introduce a novel self-powered MFC-based sensor fabricated on a single layer of paper, facilitating efficient batch manufacturing. Paper-based platforms offer numerous advantages for MFCs, including easy disposability without posing infectious health risks, inherent porosity and wickability for agent and sample delivery, and a three-dimensional environment conducive to bacterial attachment and metabolism^42–45^. Additionally, these platforms support wearability for non-invasive glucose monitoring through sweat and provide an excellent low-cost economic solution^46^. The tunability of paper with various functional materials enhances its engineering and manufacturing potential. Specifically, batch fabrication methods such as printing, screen-printing, and microfluidic injection are readily achievable. Our research group has pioneered many paper-based MFCs for various applications, demonstrating the versatility and practicality of this approach^42–46^. Here, the MFC features a horizontal configuration, integrating all components onto a single paper layer (Fig. 4a). Using a commercial wax printer, we defined component regions by printing hydrophobic wax, which functioned as the ion-exchange membrane between the anode and cathode. The wax penetrated the entire thickness of the paper, ensuring complete separation of components (Fig. 4b-i-ii). To prevent bodily fluids such as sweat from infiltrating the cathode during skin application, additional wax was applied to the bottom cathodic part, partially penetrating the paper (Fig. 4b iii). For effective harvesting of bacterial electricity, we engineered the anodic part of the paper to be conductive by introducing a water-based poly(3,4-ethylenedioxythiophene) polystyrene sulfonate (PEDOT:PSS) polymer ink. This polymer was conformally and tightly deposited on individual paper fibers, preserving the paper’s porosity. The conductivity of the polymer was enhanced with the addition of dimethyl sulfoxide (DMSO), while 3-glycidoxypropyl-trimethoxysilane (3-GLYMO) was incorporated to increase the paper’s hydrophilicity^44^. The cathodic paper part was optimized by supplementing PEDOT:PSS with Ag_2_O to enhance cathodic reduction, leveraging the stable and high-performance catalytic properties of Ag_2_O. The anodic part was pre-inoculated with *B. subtilis* endospores, providing a storable, dormant biocatalyst (Fig. S2). While previous studies demonstrated spore-forming bacterial fuel cells on paper for wearable applications^42^, their functionality was limited to one-time power generation, necessitating pre-integration of chemical germinants. In contrast, our innovative MFC design functions as a novel self-powered transducer for glucose detection, operating without the need for chemical germinants.

**Fig. 5:**
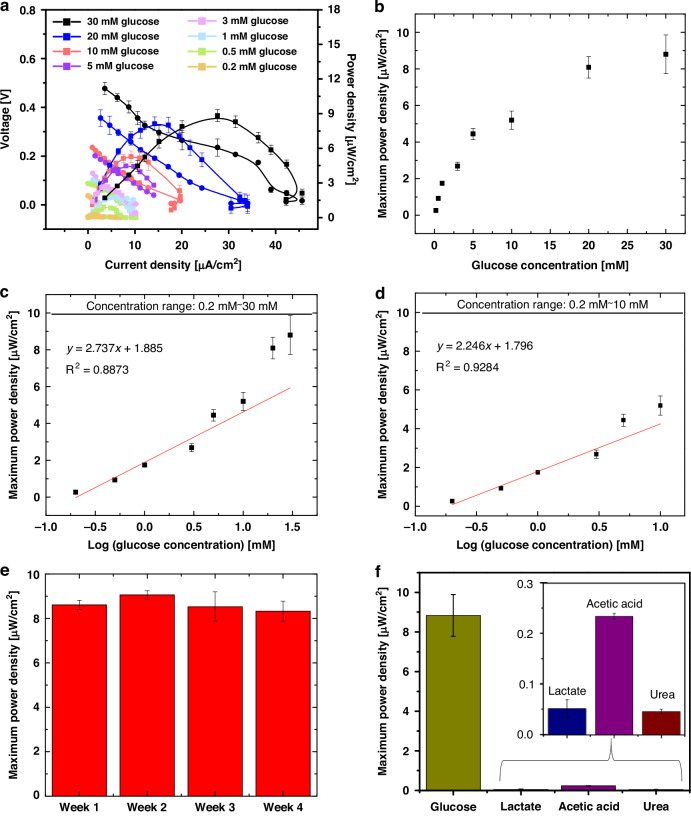
**MFC-enabled self-powered glucose monitoring**. **a** Polarization and power curves of the MFC in the presence of glucose concentrations ranging from 0.2 to 30 mM. **b** Plot of maximum power output as a function of glucose concentration. **c** Plot of maximum power output versus the logarithm of glucose concentrations from 0.2 to 30 mM. **d** Detailed plot of maximum power output versus the logarithm of glucose concentrations within the 0.2 to 10 mM range. **e** Maximum power density corresponding to each storage duration, illustrating the long-term viability of the MFC system. **f** Selectivity of the MFC-enabled glucose sensor compared to lactate, acetic acid, and urea

The MFC-based sensing capability was assessed using various glucose concentrations in a potassium-rich artificial sweat solution (Sweat Control for Cystic Fibrosis Testing-Level 1, Quantimetrix) containing 25 mM chloride, 6.7 mM potassium, and 23 mM sodium. The tested glucose concentrations ranged from 0.2 to 30 mM, reflecting the physiological levels found in blood (2–40 mM) and interstitial fluid (2–20 mM)^47^. Considering the significantly lower glucose levels in other bodily fluids, such as saliva (<2 mM) and sweat (<1 mM)^47^, concentrations below 2 mM were included in the evaluation. Specifically, the glucose concentrations tested were 30, 20, 10, 7, 5, 3, 1, 0.5, and 0.2 mM (Fig. 5a). The horizontally configured MFC was prepared as a glucose sensor by inoculating *B. subtilis* spores at an optical density (OD_600_) of 2.0, corresponding to 3.0 × 10^8^ spores per mL. Once the device was fully dried, glucose samples in artificial sweat were introduced to the anodic area of the MFC and incubated for four hours to ensure maximum spore germination. Subsequently, the polarization curve (voltage vs. current density) and power output were monitored across varying glucose concentrations. Electrical transduction signals were obtained based on the maximum voltage output with a series of external resistors (ranging from no resistor to 470, 250, 162, 100, 71, 47.5, 32, 22, 15, 10, 2, 1.5, 0.45, and 0.35 kΩ) (Fig. 5a). Maximum power outputs were generally obtained with a 15 kΩ external resistor, indicating that the internal resistance of the MFCs was approximately 15 kΩ. This resistance is comparable to that of miniature MFCs^48^, suggesting that their performance could be significantly improved by reducing the internal resistance in the future. The MFC demonstrated notable potential as a self-powered sensing device, producing sufficient electrical output to sensitively monitor various glucose concentrations in artificial sweat samples. A calibration curve was established, showing the relationship between maximum power density and glucose concentration (Fig. 5b). The power output increased linearly at lower concentrations, then non-linearly at higher concentrations until saturation, indicating maximum activity at the fixed spore concentration. To achieve a linear relationship, logarithmically transformed concentration data were applied (Fig. 5c, d)^49^. Across the entire concentration range from 0.2 mM to 30 mM, a higher sensitivity was observed, but the linear relationship was weak, reflected by a low R-squared (R^2^) value (Fig. 5c). In a selected concentration range between 0.2 mM and 10 mM, an improved R^2^ value over 0.92 was achieved, albeit with a slight sacrifice in sensitivity (Fig. 5d). Within this range, the MFC exhibited a sensitivity of 2.246 µW·(log mM)^−1^·cm^−2^ and a limit of detection (LOD) of approximately 0.07 mM. The LOD was calculated with the equation,

$${LOD}=\frac{3.3\delta }{C/p}$$

where ‘*δ’* represents the standard deviation of the power density derived from ten blank samples, ‘*C’* denotes the minimum concentration on the linear curve, and ‘*p’* indicates the power density corresponding to ‘*C’*^49^. To directly compare performance, an enzymatic fuel cell (EFC) was developed using the same device size, configuration, and materials as the MFC, with glucose oxidase (GOx) replacing the bacterial biocatalyst^14^. GOx was immobilized on the conductive PEDOT:PSS anodic reservoir using a solution of graphene and chitosan^50^. Chitosan has a strong affinity for enzymes, while graphene nanoparticles act as mediators, transferring electrons to the anode. Like the MFC, the EFC functions as a self-powered glucose sensor, generating an electrical signal when GOx catalyzes glucose oxidation^50,51^. The EFC’s electrical output increases as glucose concentration increases (Fig. S3a). The calibration curve is non-linear (Fig. S3b), similar to the MFC, but the relationship between power output and the logarithm of glucose concentration becomes linear (Fig. S3c)^49^. The EFC’s sensitivity for glucose detection is 3.61 µW·(log mM)^−1^·cm^−2^, with a LOD of approximately 0.02 mM, slightly better than the MFC. However, considering the EFC’s generally higher power density because of a lower energy barrier for electron transfer, the comparable performance of our MFC is particularly notable. Especially in terms of shelf-life, the MFC’s performance remains exceptional. The EFC’s power density gradually decreases with daily storage at room temperature and significantly declines weekly (Fig S3d, e). After four weeks, the EFC loses more than 85% of its initial power (Fig. S3f). In contrast, the MFC maintains its performance after four weeks of storage, even under non-optimized environmental conditions (Fig. 5e, S4a, b). This highlights the MFC’s exceptional stability and longevity, establishing it as a very reliable choice for long-term applications. The dormancy of *B. subtilis* effectively preserves bacterial viability, granting the system an impressive shelf-life in its metabolically inactive spore state^40^. Because of their minimal water content and multiple protective layers, *B. subtilis* spores can remain dormant for extended periods, often spanning many years^28^. Those dormant spores exhibit remarkable resistance to extreme conditions, including desiccation, heat, radiation, toxicants, and nutrient deprivation, ensuring they maintain viability even under suboptimal storage conditions^27^. Upon detecting specific nutrient signals, such as glucose, the endospores exit dormancy and resume metabolic activity.

The MFC-based sensor demonstrated exceptional selectivity, effectively distinguishing glucose even in the presence of various interferents such as lactate, acetic acid, and urea (Fig. 5f). When compared to the current response to glucose (30 mM), these interferents (30 mM each) produced negligible signal changes. This high selectivity is attributed to the specificity of spore germination to glucose in potassium-rich fluids and their active metabolism of glucose. Consequently, our MFC-based sensor demonstrated excellent selectivity for glucose.

### Continuous and sustained glucose monitoring through cyclical sporulation and germination

**Fig. 6:**
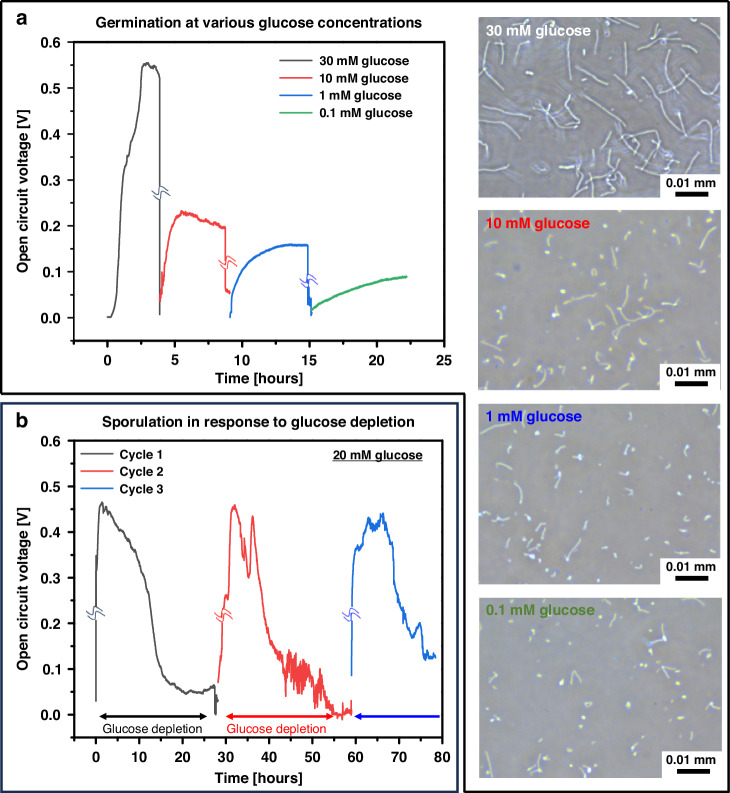
**Continuous and sustained glucose monitoring via cyclical sporulation and germination**. **a** Real-time open circuit voltage (OCV) of the MFC during cyclic germination and sporulation under varying glucose concentrations, accompanied by visual images capturing spore germination. The data emphasizes the germination phase by omitting details of the sporulation process for clarity. **b** OCV of the MFC during cycles of germination under 20 mM glucose concentration, with a focus on the sporulation phase, omitting the germination process for a clearer analysis of sporulation dynamics

Our microbial sensing platform presents a compelling alternative to traditional enzymatic glucose sensors, particularly in addressing the limitations associated with enzyme degradation through time. Unlike enzymatic sensors, our microbial sensors leverage the ability of living bacteria to undergo cyclical sporulation and germination in response to glucose, thereby continuously replenishing active enzymes and ensuring long-term stability. By continuously monitoring the open circuit voltage (OCV) of the MFC, we observed that the cyclical processes of germination and sporulation are closely linked to glucose concentration (Fig. 6a). Higher glucose concentrations led to increased OCV values, indicative of a greater number of germinated cells actively participating in metabolic processes. In contrast, lower glucose concentrations resulted in lower OCV as germination remained in its early stages, as confirmed by microscopic analysis. Crucially, during these repeated germination cycles, the MFC consistently produced reliable and stable OCV values, in agreement with the outputs depicted in Fig. 5a. This consistency underscores the robustness of the microbial sensor, particularly in maintaining performance for extended periods. Lower glucose concentrations required more time to achieve stable OCV values—0.1 mM glucose took more than 8 h to stabilize, while 10 mM glucose required only three hours. This time-dependent behavior emphasizes the strong relationship between glucose concentration, spore germination propensity, and outgrowth rate.

We evaluated the repeatability and stability of the MFC during multiple cycles of glucose depletion and reintroduction (Fig. 6b). Following the introduction of 20 mM glucose, the MFC generated an OCV of approximately 0.45 V. As glucose was depleted, the bacterial cells transitioned into their dormant spore state, resulting in a significant drop in OCV to nearly zero because of the cessation of metabolic and electrogenic activities. Remarkably, upon reintroduction of glucose, the MFC quickly restored a similar OCV, demonstrating the system’s resilience and reproducibility. These findings highlight the potential of our spore-forming bacterial sensing system as a reliable, stable, and reproducible platform for CGM. By harnessing the natural life cycle of bacteria, this approach could pave the way for next-generation glucose monitoring systems that overcome the limitations of conventional enzymatic sensors, offering sustained and robust performance for long-term applications.

### Integration with portable readout interface for potential wearable applications

**Fig. 7:**
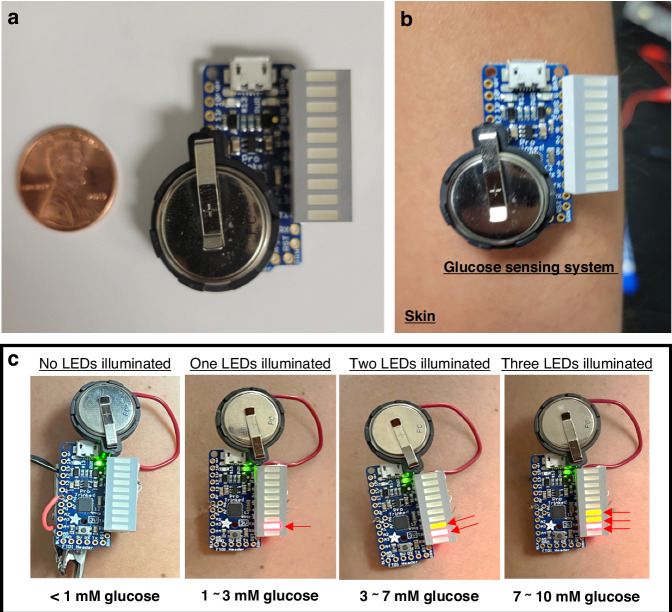
**Integration of the MFC-based glucose sensor with a portable readout interface for potential wearable applications**. **a** The compact MFC-based glucose sensing system shown alongside a 1 US cent coin for size comparison. **b** Demonstration of the wearable feature, highlighting its potential for non-invasive glucose monitoring. **c** Optical outputs of the glucose sensing system corresponding to different glucose levels: less than 1 mM, 1–3 mM, 3–7 mM, and 7–10 mM

While our MFC-based glucose sensor is inherently self-powered, generating its own transducing output signal, a suitable readout instrument is necessary to visualize and interpret this signal effectively. A widely available and cost-effective digital multimeter can be employed for this purpose^50^. However, its bulky design and lack of integration with the sensor limit its portability and user-friendliness, thereby restricting its potential applications in settings where compactness and ease of use are critical. In this study, we successfully integrated our MFC sensor into a compact and wearable readout interface, designed to provide real-time visual alerts for glucose monitoring. The system used a segmented LED array to display glucose concentration levels, with the voltage output from the MFC being directly proportional to the glucose levels present in the sample (Fig. 7a). The voltage generated by the MFC was interpreted by a microcontroller, powered by a coin cell battery (Fig. S5), which then translated the data into a visual output via the LED array. The microcontroller was meticulously programmed to correlate specific voltage readings to predefined glucose concentration thresholds. This design allows the system to accurately reflect glucose levels through the activation of LEDs. For instance, when glucose levels were below 1 mM, no LEDs were illuminated, signifying low glucose concentration (Fig. S6a). At glucose concentrations from 1 to 3 mM, corresponding to typical levels found in normal blood samples, one LED was activated (Fig. S6b). As glucose levels approached the prediabetic range of 3–7 mM, two LEDs were illuminated, indicating a potential risk for developing diabetes (Fig. S6c). Finally, when glucose levels exceeded 7 mM, up to three LEDs were activated, serving as a critical alert for diabetes (Fig. S6d). To ensure the practicality of the system, we designed it to be compact and wearable, employing 3 M Tegaderm medical transparent tape to adhere to the skin (Fig. 7b). The device’s wearability and functionality were validated through tests with pre-loaded glucose concentrations, demonstrating reliable and consistent performance (Fig. 7c). The integration of the MFC sensor into a wearable interface marks a significant advancement in non-invasive glucose monitoring technology. By harnessing MFC technology, the system’s design is notably streamlined, eliminating the need for complex signal transduction. This simplification reduces the system’s complexity and enhances its reliability. The LED-based visual readout provides an intuitive and immediate display of glucose levels, a feature particularly crucial for effective diabetes management. The system’s capability to accurately detect glucose levels within clinically relevant ranges establishes it as a reliable tool for prediabetic and diabetic patients. Its compact, wearable design, combined with minimal power requirements, ensures that it can be seamlessly integrated into daily life. That allows for CGM with minimal discomfort without frequent maintenance, making it a practical and user-friendly solution for long-term health management.

## Future direction

Here, highly selective and sensitive glucose detection was achieved by creating a novel transducing mechanism based on bacterial germination. However, its practical application requires further investigation. The time needed for germination poses a significant hurdle for rapid diagnostics and remains a significant challenge. In our study, the majority of spores required more than one hour to reach maximum germination and power output, with full performance achieved only after four hours. This delay highlights a critical limitation in the current design, as rapid diagnostics are essential for real-time monitoring and clinical applications. Previously, we demonstrated that leveraging heat activation could revolutionize the germination process^40^. By applying heat treatment, we were able to induce full germination and achieve maximum power output within just tens of minutes—a significant improvement over the natural germination process. This suggests that heat treatment could be a viable strategy for accelerating germination, enabling timely diagnostics^52,53^. Future studies should focus on optimizing this technique, potentially exploring different temperatures and durations to enhance germination speed without compromising sensor accuracy.

Another critical area that warrants exploration is the effect of potassium concentration in bodily fluids on germination^32,33^. As discussed, glucose-triggered germination is very sensitive to potassium levels, which vary between individuals and can fluctuate in different physiological conditions^54^. Currently, research is scarce on the relationship between the potassium-glucose concentration ratio and bacterial germination. Understanding this relationship is essential for the practical application of our spore-forming MFC-based glucose sensor, as variations in potassium levels could lead to inconsistencies in sensor performance. Future research should focus on addressing these challenges by optimizing germination conditions and thoroughly investigating the effect of bodily fluid composition on sensor performance. By overcoming those hurdles, we can move closer to developing a reliable, rapid, and practical glucose sensor for clinical applications.

Lastly, while our microbial system has shown impressive selectivity and longevity, its sensitivity at lower glucose concentrations (0.2–1.0 mM) remains slightly lower than traditional enzymatic systems. To address this, we are actively working on improving the electrode design and optimizing the MFC setup.

## Conclusion

While CGM systems have advanced, offering real-time data and reduced invasiveness compared to traditional finger-prick methods, their reliance on enzymatic sensors limits their operational lifespan and stability from enzyme denaturation. Addressing that limitation, this study introduced an innovative, spore-forming microbial whole-cell sensing system for glucose detection. Leveraging the germination response of *B. subtilis* spores to glucose in potassium-rich environments, the system initiates metabolic activity that generates measurable electrical signals within a paper-based MFC. This approach extended shelf-life through the dormant spore state and ensured robustness via the self-replicating nature of the bacteria upon germination. The MFC demonstrated high sensitivity and selectivity to glucose concentrations ranging from 0.2 to 10 mM, with a notable limit of detection at approximately 0.07 mM. Comparative analyses revealed that, unlike conventional enzymatic biosensors that suffer significant performance degradation over time, the spore-based MFC maintained stable functionality for extended periods. Integration with a compact, wearable readout interface showcased the system’s potential for non-invasive, CGM applications. From a commercial and application perspective, our approach has the potential to revolutionize wearable glucose sensors by enabling long-lasting, low-maintenance devices with extended shelf lives. Despite challenges related to slow germination and the influence of potassium concentration on its practical use, our spore-forming microbial whole-cell sensing strategy developed a cost-effective, stable, and sustainable alternative for glucose monitoring. The approach not only addresses critical limitations of existing technologies but lays the groundwork for advanced biosensing applications, offering significant potential for future innovation in the field.

## Materials and methods

### Materials

Whatman™ Filter Paper (3MM) was sourced from Sigma-Aldrich, while Graphite Ink (E3449) was obtained from Fisher Scientific. Additional reagents including Sodium Chloride (NaCl), Agar, Tryptone, Yeast Extract, Carboxyfluorescein Diacetate (cFDA), Propidium Iodide (PI), sodium dodecyl sulfate (SDS), Dimethyl Sulfoxide (DMSO), 3-GLYMO, Phosphate Buffer Saline (PBS), Potassium Ferrocyanide (K_4_[Fe(CN)_6_]), Glucose Oxidase (GOx) from Aspergillus niger, Chitosan, Acetic Acid, Urea, Sodium L-Lactate, and D-Glucose were all procured from Sigma-Aldrich. Poly(3,4-ethylenedioxythiophene): Polystyrene Sulfonate (PEDOT: PSS; Clevios PH1000) was supplied by Heraeus, Germany. Silver Oxide (Ag_2_O) was acquired from Alfa Aesar.

### Bacterial inoculum and sporulation

The *Bacillus subtilis* strain 168, sourced from the American Type Culture Collection (ATCC), was initially cultured in Luria Broth (LB) medium and incubated at 37 °C for 24 h. Post-incubation, the bacterial cells were harvested via centrifugation and subjected to serial dilution for subsequent experiments. The concentration of *B. subtilis* was monitored by measuring the optical density at 600 nm (OD_600_), with the cell pellet resuspended in fresh LB to achieve an OD_600_ of 2.0. To induce sporulation, the bacterial culture was transferred to nutrient-depleted agar plates. The resulting spores were collected and centrifuged at 4000 rpm for 4 min. The spore pellet was then resuspended in distilled water and subjected to heat treatment at 80 °C for 30 min to eliminate any remaining vegetative cells.

### Bacterial spore purification

Spore purification was performed using a modified version of Tavares’ and Souza’s second purification method^55^. The spores, suspended in distilled water, were centrifuged at 4000 rpm for 10 min. The supernatant was carefully removed using a micropipette, and the spores were resuspended in PBS containing 50 μg/mL lysozyme. After vortexing, the suspension was incubated on a shaker at 37 °C for 1 h. The spores were then washed with distilled water and resuspended in a 0.05% SDS solution. Following three additional rinses with distilled water, the spores from eight agar plates were pooled, yielding approximately 1 mL of purified spores per tube. These spores were then stored as a pellet or resuspended in 2 mL of deionized water for application onto the device anode.

### Fabrication of the MFC

A sheet of Whatman 3 MM CHR filter paper was first patterned with hydrophobic wax boundaries using a Xerox Phaser ColorQube 8570 commercial wax printer, with the wax applied to both sides of the paper. Thermal treatment at 150 °C for 30 seconds was then used to define the front side, containing the anode and cathode, and the back side, with the cathode section blocked. The anodic electrolyte, consisting of a mixture of 2 mL PEDOT:PSS and 10 μL DMSO, and the cathodic electrolyte, comprising 300 mg of Ag_2_O in 10 mL PEDOT:PSS, were applied to their respective areas. To enhance hydrophilicity and effectively preload the aqueous bacterial culture, 2 wt% 3-GLYMO was added to the anodic region. Finally, graphite was screen-printed onto both compartments using a sacrificial mask to serve as the current collector.

### Fabrication of the EFC

EFCs and MFCs are both biofuel cells that generate electricity, which in this study can be harnessed as a sensing signal for glucose detection. However, they differ significantly in their catalysts and fabrication processes. EFCs use isolated enzymes as biocatalysts, which can degrade or lose activity over time. In contrast, MFCs rely on whole microorganisms that can adapt to varying conditions and sustain long-term operation. The fabrication of EFCs typically involves immobilizing enzymes onto the electrode surface, requiring careful optimization of enzyme orientation and proximity to the electrode to enhance electron transfer efficiency. Below is a detailed description of the EFC fabrication process. The fabrication of the wax patterns for the anode, cathode, and separator, along with the conductive engineering of the electrodes in the EFC, was executed using methods akin to those employed for MFCs. Following this, a 32 mL aliquot of GOx solution was uniformly applied to the anode. To ensure the effective immobilization of GOx on the conductive anodic reservoir, a graphene-chitosan solution was first coated onto the anode^50^. The solution was created by sonicating 1 mL of 0.5 wt% chitosan in 2% acetic acid for 15 min, after which 1 mg of graphene powder (Angstron Materials, Dayton, OH, USA) was added. The resulting graphene/chitosan composite provided a biocompatible matrix ideal for immobilizing the redox enzyme, GOx, because of chitosan’s excellent film-forming properties and its ability to disperse nanomaterials effectively. The graphene nanoparticles within the matrix acted as mediators, enabling direct electron transfer from GOx to the anode, which in turn improved the stability and sensitivity of glucose detection.

### Electrochemical and electrical characterization

Electrochemical measurements, including cyclic voltammetry (CV) and electrochemical impedance spectroscopy (EIS), were conducted using a Squidstat Plus potentiostat (Admiral Instruments). The experiments utilized a screen-printed electrode provided by Metrohm, USA, and were carried out in a K_4_[Fe(CN)_6_]/PBS buffer solution. CV was employed to explore the redox characteristics and charge transfer properties of the materials, offering insights into their electrochemical behavior. EIS, conducted in PBS, was used to evaluate the impedance properties across a spectrum of frequencies, revealing the materials’ resistive and capacitive behaviors. EIS data was analyzed and modeled using ZView® 4.0 software. The electrical performance of the MFCs and EFCs was assessed through a data acquisition system (DATAQ Instruments) connected to various external resistors. Polarization curves and power outputs were derived by measuring voltage drops across these resistors, enabling the calculation of current and power. Power and current densities were normalized to the anodic surface area.

### Integration of the MFC with a portable readout interface

Initially, the pins of the 10-segment LED array (KINGBRIGHT DC-10YWA) were identified and mapped to the corresponding digital pins on the microcontroller (ATmega328P 3 V/8 MHz - DEV-11113). Specifically, each segment of the LED array was connected to a designated digital pin on the microcontroller, such as Segment A to Digital Pin 2, Segment B to Digital Pin 3, and so forth. The circuit was powered by a CR2025 coin cell battery, with its positive terminal connected to the VCC pin of the microcontroller and the negative terminal to the GND pin. Additionally, each LED segment was properly grounded. The MFC, functioning as a biosensor, generates a voltage proportional to microbial activity, which correlates with glucose concentration. This voltage output from the MFC is fed into the microcontroller, which is powered by the coin cell battery. The microcontroller is pre-programmed to process the voltage readings from the MFC and translate them into a visual output on the 10-segment LED array. The LED array is configured such that the number of illuminated segments directly corresponds to the voltage level detected, with higher voltages resulting in a greater number of segments lighting up. This provides a visual representation of glucose concentration, where the intensity of the LED response indicates the glucose level detected by the MFC.

### Statistical analysis

Statistical analysis was conducted on experimental data obtained from a minimum of three identical trials. The results are presented as the mean ± standard error of the mean for these replicates, providing a robust measure of variability and accuracy.

## Supplementary information

Supporting Information
