# Supplementary material for: Revolutionary self-powered transducing mechanism for long-lasting and stable glucose monitoring: achieving selective and sensitive bacterial endospore germination in microengineered paper-based platforms
Source: Microsyst Nanoeng. 2024 Dec 12;10:187. doi: 10.1038/s41378-024-00836-9 (PMC11634959; doi:10.1038/s41378-024-00836-9)

Supporting Information

**Revolutionary Self-Powered Transducing Mechanism for Long-Lasting and Stable Glucose Monitoring: Achieving Selective and Sensitive Bacterial Endospore Germination in Microengineered Paper-based Platforms**

Yang Gao^1^, Anwar Elhadad^1^, and Seokheun Choi^1,2^*

^1^Bioelectronics & Microsystems Laboratory, Department of Electrical & Computer Engineering, State University of New York at Binghamton, Binghamton, New York, 13902, USA

^2^Center for Research in Advanced Sensing Technologies & Environmental Sustainability, State University of New York at Binghamton, Binghamton, New York, 13902, USA

*Corresponding Author. Email: [sechoi@binghamton.edu](mailto:sechoi@binghamton.edu)

Lab website: <http://ws.binghamton.edu/choi/>

Center website: <http://www.ws.binghamton.edu/creates/>

Table S1, calculated resistance of the equivalent circuit using ZView software.

| **Glucose Concentration (mM)** | \|  \| \| --- \|   **Rct (Ω)** |
| --- | --- | --- |
| 0 mM (Control) | 700 |
| 0.2 mM | 450 |
| 1 mM | 350 |
| 5 mM | 250 |
| 10 mM | 200 |

Figure S1. Germination of *B. subtilis* spores in response to 0.2 mM glucose. (a) Brightfield microscopic image of the spores, illustrating their morphological state, and (b) corresponding fluorescent image, highlighting active germination sites through specific fluorescence markers.


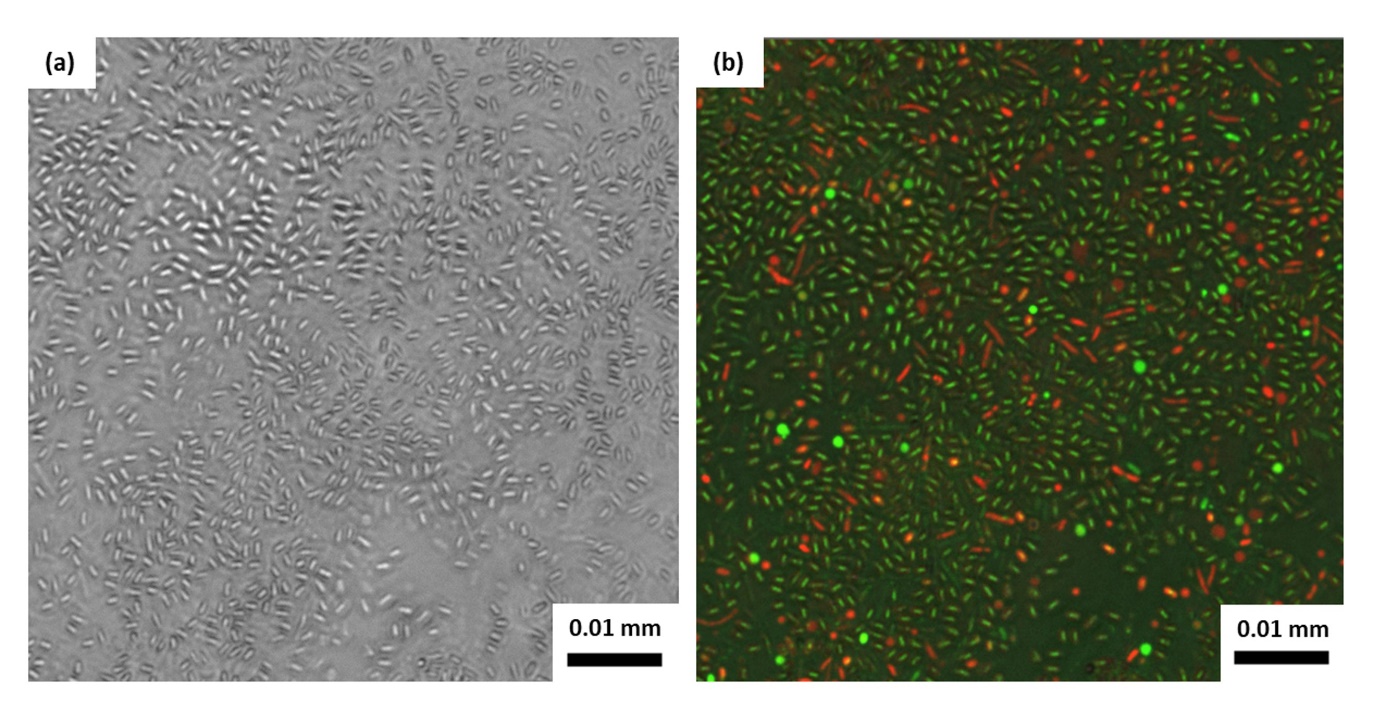


Figure S2. SEM image of *Bacillus subtilis* spores pre-inoculated in the anodic reservoir, showcasing their surface morphology and spatial distribution prior to germination.


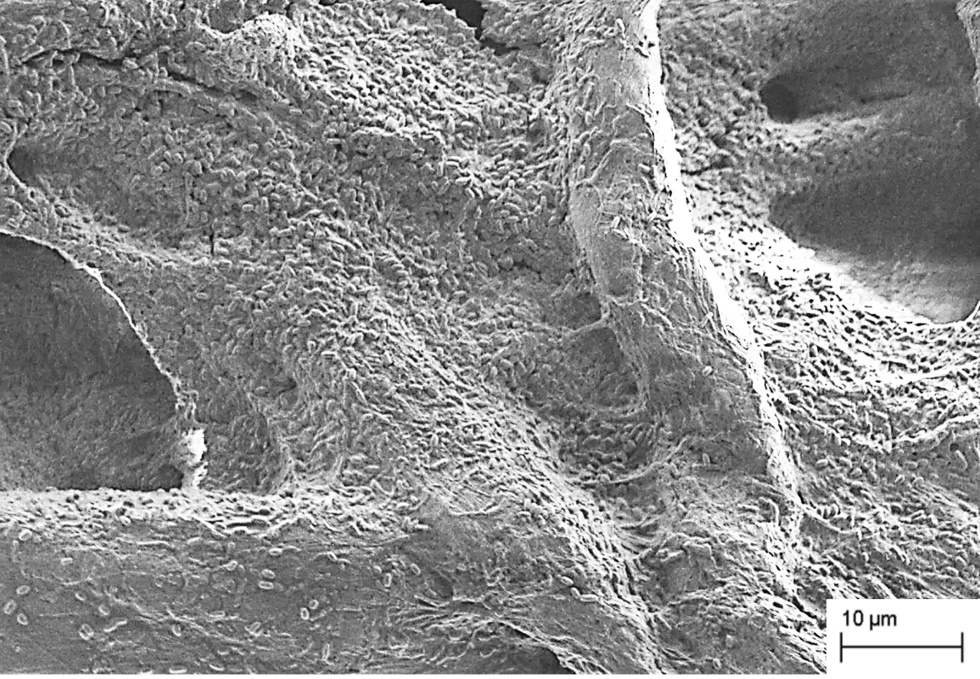


Figure S4. The effect of storage duration on performance of the MFC-enabled self-powered glucose monitoring. (a) Polarization curve and power output of the MFC measured daily over a seven-day period, indicating stability over time. (b) Polarization curve and power output of the MFC after 1, 2, 3, and 4 weeks of storage, showing the effect of storage duration on performance.


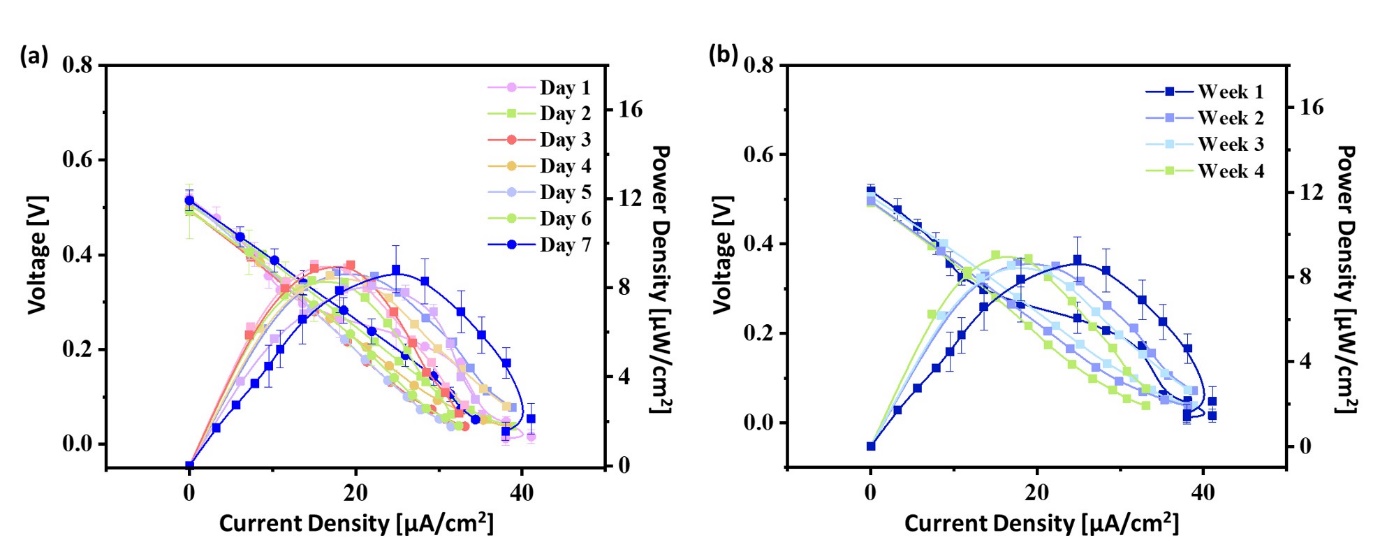


Figure S3. EFC-enabled self-powered glucose monitoring. (a) Polarization and power curves of the EFC in response to glucose concentrations ranging from 0.2 to 30 mM. (b) Maximum power output plotted as a function of glucose concentration. (c) Maximum power output versus the logarithm of glucose concentrations, highlighting the linearity of the response. (d) Polarization curve and power output of the EFC measured daily over a seven-day period. (e) Polarization curve and power output of the EFC after 1, 2, 3, and 4 weeks of storage. (f) Maximum power density corresponding to each storage duration.


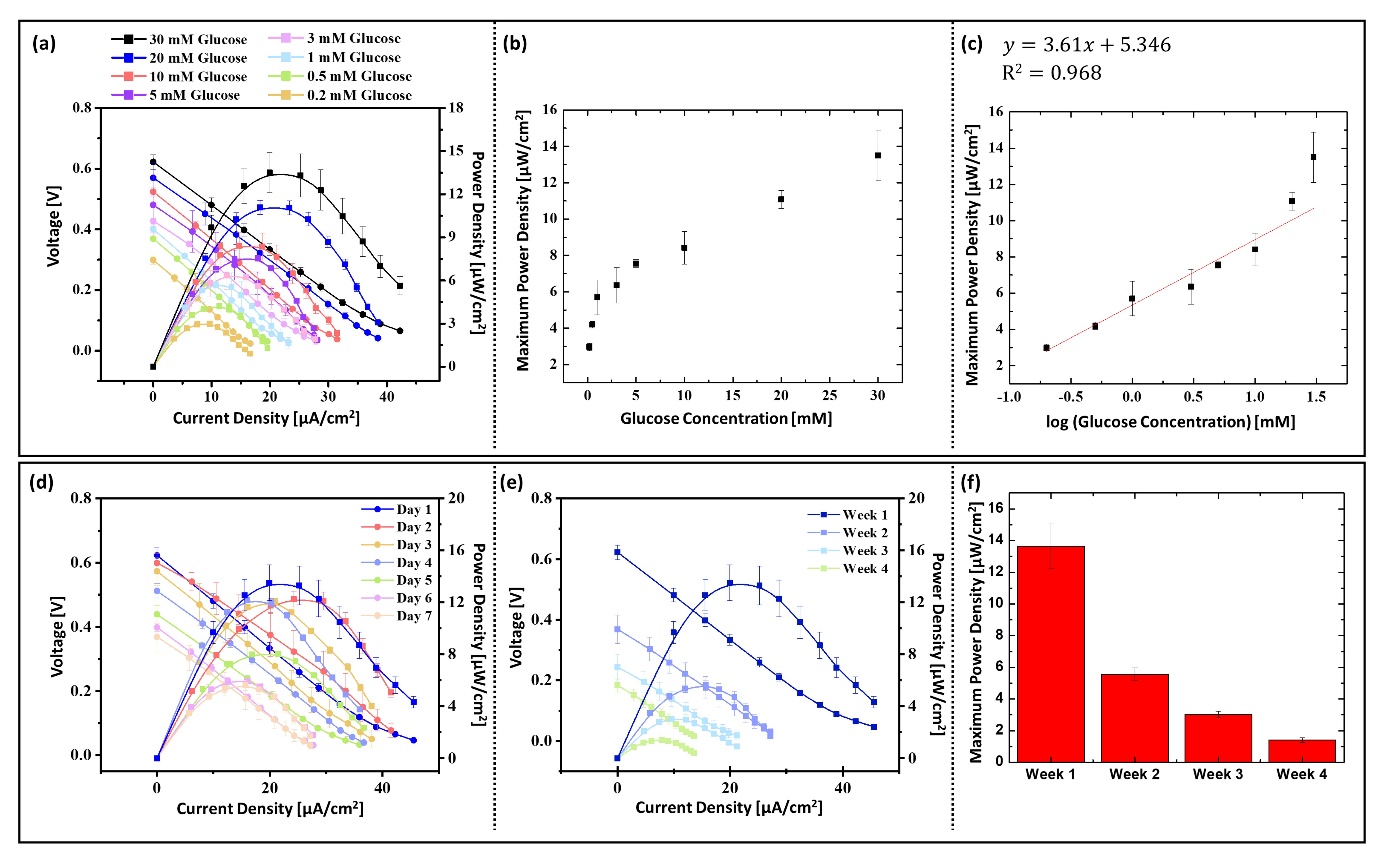


Figure S5. Integration of the MFC with the readout interface, including a detailed schematic of the microcontroller connections to various components.


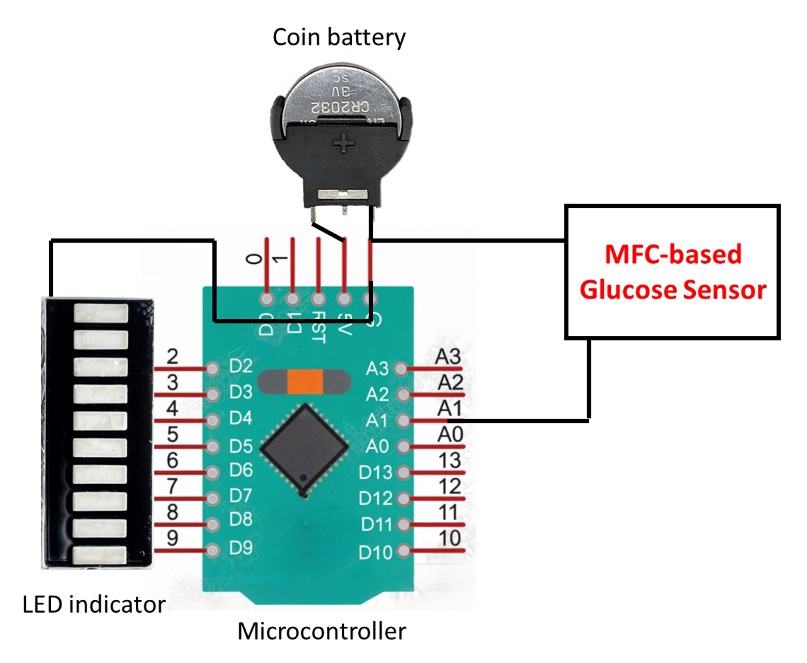


Figure S6. Optical outputs of the glucose sensing system, where each LED indicator in the array corresponds to a specific glucose concentration. LED responses are shown for different glucose levels: (a) less than 1 mM, (b) 1-3 mM, (c) 3-7 mM, and (d) 7-10 mM.


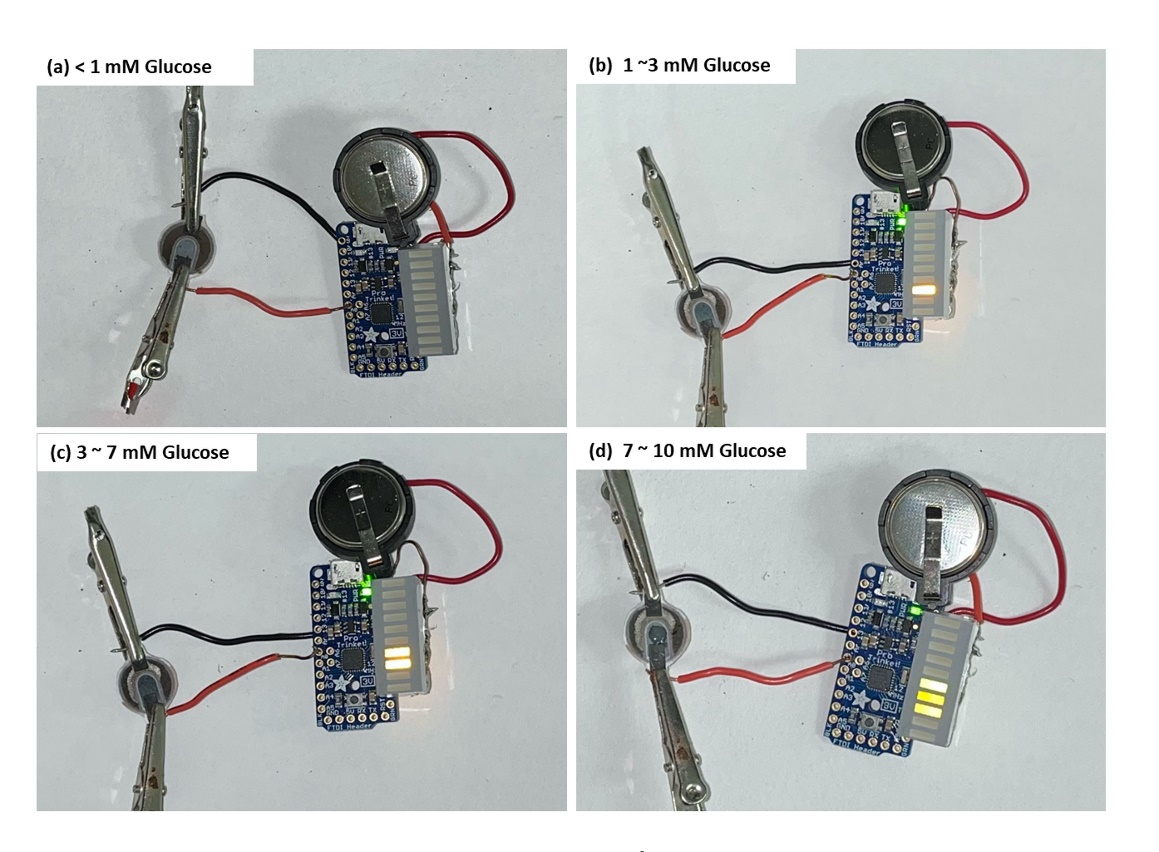

Supplement: Supplementary file 1 — Supporting Information [file 41378_2024_836_MOESM1_ESM.docx]
